# Socioeconomic, hygienic, and sanitation factors in reducing diarrhea in the Amazon

**DOI:** 10.1590/S1518-8787.2016050006505

**Published:** 2016-11-24

**Authors:** Katiuscia Shirota Imada, Thiago Santos de Araújo, Pascoal Torres Muniz, Valter Lúcio de Pádua

**Affiliations:** I Centro de Ciências da Saúde e do Desporto. Universidade Federal do Acre. Rio Branco, AC, Brasil; II Departamento de Engenharia Sanitária e Ambiental. Escola de Engenharia. Universidade Federal de Minas Gerais. Belo Horizonte, MG, Brasil

**Keywords:** Child, Preschool. Diarrhea, epidemiology, Basic Sanitation, Water Supply, Domestic Effuents, Community Development, Socioeconomic Factors

## Abstract

**OBJECTIVE:**

To analyze the contributions of the socioeconomic, hygienic, and sanitation improvements in reducing the prevalence of diarrhea in a city of the Amazon.

**METHODS:**

In this population-based cross-sectional study, we analyzed data from surveys conducted in the city of Jordão, Acre. In 2005 and 2012, these surveys evaluated, respectively, 466 and 826 children under five years old. Questionnaires were applied on the socioeconomic conditions, construction of houses, food and hygienic habits, and environmental sanitation. We applied Pearson’s Chi-squared test and Poisson regression to verify the relationship between origin of water, construction of homes, age of introduction of cow’s milk in the diet, place of birth and the prevalence of diarrhea.

**RESULTS:**

The prevalence of diarrhea was reduced from 45.1% to 35.4%. We identified higher probability of diarrhea in children who did not use water from the public network, in those receiving cow’s milk in the first month after birth, and in those living in houses made of *paxiúba*. Children born at home presented lower risk of diarrhea when compared to those who were born in hospital, with this difference reversing for the 2012 survey.

**CONCLUSIONS:**

Sanitation conditions improved with the increase of bathrooms with toilets, implementation of the *Programa de Saúde da Família* (PSF – Family Health Program), and water treatment in the city. The multivariate regression model identified a statistically significant association between use of water from the public network, construction of houses, late introduction of cow’s milk, and access to health service with occurrence of diarrhea.

## RESUMO

**OBJETIVO:**

Analisar as contribuições das melhorias socioeconômicas, higiênicas e de saneamento na redução da prevalência de diarreia em uma cidade na Amazônia.

**MÉTODOS:**

Neste estudo transversal de base populacional, foram analisados dados dos inquéritos realizados no município de Jordão, Acre. Em 2005 e 2012, foram avaliadas, respectivamente, 466 e 826 crianças menores de cinco anos. Foram aplicados questionários sobre as condições socioeconômicas, construção dos domicílios, hábitos higiênicos e alimentares e saneamento ambiental. Foi aplicado o teste Qui-quadrado de Pearson e a Regressão de Poisson para verificar a relação existente entre procedência da água, tipo de construção do domicílio, idade de introdução de leite de vaca na dieta e local de nascimento e a prevalência de diarreia.

**RESULTADOS:**

A prevalência de diarreia foi reduzida de 45,1% para 35,4%. Foi identificada maior probabilidade de desenvolvimento de diarreia em crianças que não utilizaram água da rede pública, as que receberam leite de vaca no primeiro mês após o nascimento e as residentes em domicílios de paxiúba. As crianças que nasceram no domicílio apresentaram menor risco de diarreia quando comparadas às que nasceram em hospital, com essa diferença se invertendo para o inquérito de 2012.

**CONCLUSÕES:**

Ocorreu melhora nas condições de saneamento com aumento no número de banheiro com vasos sanitários, implantação do Programa de Saúde da Família e tratamento de água na sede do município. O modelo de regressão multivariada identificou associação estatisticamente significativa entre utilização de água da rede pública, construção da moradia, introdução tardia de leite de vaca e acesso a serviço de saúde com ocorrência de diarreia.

## INTRODUCTION

Sanitation, recognized as an important health promotion strategy, figures on the international agenda among the eight Millennium Development Goals, whose aim to halve the population without access to drinking water was reached in advance. However, 11.0% of the world’s population still remains without access to drinking water. Overcoming this challenge requires facing technological, political, and managerial barriers, which are even more complex in the Amazon because of natural obstacles.

In Brazil, basic sanitation is a right ensured by the Constitution and defined by the Law 11,445/2007, which establishes the national guidelines for sanitation, defined as the set of services, infrastructure, and facilities of water supply, sewage system, urban cleaning, urban drainage, solid waste and rainwater management^a^. These services promote the improvement of the quality of life of the population, reflecting directly on child health, with reduction of child mortality and of diarrheal, parasitic, and skin diseases. Besides, they protect the health of the population, minimizing the consequences of poverty and also protecting the environment^b^.

Diarrhea is a serious public health problem related to hygiene conditions and quality of water used^9^. It is the second most common cause of childhood deaths, representing around 1.5 million deaths/year in children under five years^c^. There are many determinant factors^13^; about 88% of diarrhea deaths are attributed to unsafe drinking water, inadequate sewage system, and precarious hygiene^c^. Thus, measures to prevent infant diarrhea must be prioritized as key strategy for improving child health. Among the measures, are the following: provision of water, both in quantity and quality; removal and treatment of domestic sewage; and promotion of sanitation actions in all community^c^. The infrastructure sector must supply treated water, sewage system, solid waste cleaning and collection, and rainwater drainage and management, with participation of the public health sector and community, as foreseen in the Federal Constitution of 1988^d^.

In some isolated and poorer localities of the State of Acre, as is the case of Jordão, the expansion of the supply network of drinking water and sewage system and also of medical care occurred slowly because of logistical difficulties, especially for the rural population. This study aimed to analyze the contribution of sanitation actions such as drinking water supply, construction of houses, introduction of cow’s milk, and place of birth on the reduction of the prevalence of diarrhea in children under five in the urban and rural area of the city.

## METHODS

This population-based cross-sectional study used data from two surveys conducted in the city of Jordão, Acre, in the years of 2005 and 2012, which evaluated, respectively, 466 and 826 children under five in the urban and rural area^8^.

Jordão is located at 700 km from Rio Branco, in the State of Acre, and was created in 1992, after separation from the city of Tarauacá, located in Vale do Juruá. It has an area of 5,429 km2, bathed by the rivers Jordão and Tarauacá, and borders the cities of Marechal Thaumaturgo, Feijó, and Tarauacá, and the country Peru. The population of the city in the 2010 Census was of 6,577 inhabitants, with 2,272 in the urban area and 4,305 in the rural area^e^. It is one of the most isolated cities of the State for having access only by water and air, contributing to the maintenance of high prices of goods and food. Until 2005, it did not have water treatment nor sewage system.

In the context of Amazonian and Acre reality, Jordão is considered one of the poorest cities of the Country. In 2000, its Human Development Index (HDI) was of 0.222 and, in 2010, it increased to 0.469^f^. However, this is still considered a very low HDI, figuring among the 10 lowest of Brazil.

In both surveys, the information of the characteristics of houses, environmental sanitation, and health were obtained by questionnaires, conducted by properly trained researchers. The respondent was an adult responsible by the home, who was asked to characterize the occurrence of diarrhea and whether the child(ren) of the house presented in the last 15 days more than four bowel movements/day of liquid consistency or with increase in volume. For the socioeconomic assessment, we evaluated the presence of electrical energy; benefit from *Bolsa Família* Program; maternal education; and household income. Regarding the houses, we considered the type of construction material of the house, roof, and floor, and the total rooms. Concerning infant feeding, we considered the period of introduction of cow’s milk. About the place of birth, we considered whether it occurred at home or in hospital. To evaluate environmental sanitation, we considered the type of toilet in the home and the destination of trash, as well as the origin, frequency of lack, and type of treatment carried out on drinking water.

For the categorical variables, we applied Pearson’s Chi-squared test and linear trend test, which consider the exposure level at the moment of identifying differences between the two surveys. To obtain the crude and adjusted association measures and assessment of the association strength between independent variables and outcome, we used Poisson regression with robust error estimation (Stata 9.0).

The projects were approved by the Research Ethics Committees of Fundação Hospitalar Estadual do Acre and Universidade Federal do Acre (UFAC) (Opinions 042/2005 and 23107.017408/2010-16). The guardians of the children signed the informed consent form.

## RESULTS

Comparing the 2005 and 2012 surveys, the prevalence of diarrhea decreased from 45.1% (95%CI 40.5–49.7) to 35.4% (95%CI 32.1–38.7), respectively (Figure). This reduction was expressive in the urban area. Regarding hospitalizations by diarrhea, there was an increase from 4.5% to 10.7% between 2005 and 2012. At the same time, the rate of hospitalization by diarrhea increased in the urban area of the city (p < 0.001).

**Figure:**
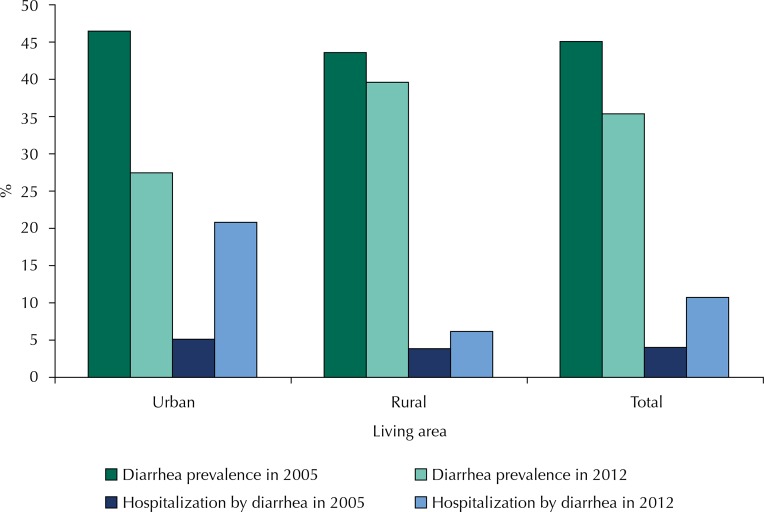
Prevalence of diarrhea in past 15 days and hospitalization by this harm in children of the urban and rural area of the city of Jordão, Acre, 2005 and 2012.

Table 1 presents data on sociodemographic and environmental conditions for the years 2005 and 2012. There was no change in the proportion of families with children under five years with access to electrical energy and in the proportion of minimum wages received by households. However, the proportion of families benefited by the Family Allowance Program increased, rising from 23.9% in 2005 to 53.9% in 2012. The maternal education level increased, reducing illiteracy in 5.4 percentage points. We observed a significant reduction, especially in the urban area, of homes built with *paxiúba* ^g^, as well as increased use of wood in the construction of houses and on floors. Considering the type of roof of the houses, straw was the material more employed. There was no significant increase in the number of rooms of the houses. Regarding the place of birth of the children, there was a reduction of births in hospital and increase in home childbirth. The introduction of cow’s milk on infant feeding in the first month of life increased, with values of 43.0% and 24.8% in 2005 and 2012, respectively.

**Table 1:** Socio-environmental characteristics of houses, place of birth, and introduction of cow’s milk in the diet of children under five years old living in the urban and rural area of the city of Jordão, Acre, 2005 and 2012.

Variable	2005 survey		2012 survey		p
	
	n	%	n	%	
Electrical energy					0.304
Yes	160	35.2	303	38.3	
No	295	64.8	489	61.7	
Total	455	100	792	100	
Family Allowance Program					< 0.001
Yes	110	23.9	437	53.9	
No	351	76.1	373	46.1	
Total	461	100	810	100	
Mother’s education level					0.017
Illiterate	79	17.4	99	12.0	
1-4 years	214	47.0	411	49.8	
5-8 years	96	21.2	186	22.5	
9-11 years	57	12.6	96	11.6	
≥ 12 years	8	1.8	34	4.1	
Total	454	100	826	100	
Household income (minimum wages)					0.545
0-0.5	191	41.8	367	45.2	
0.5-0.9	88	19.3	138	17.0	
1-1.9	122	26.7	219	27.0	
≥ 2	56	12.5	88	10.8	
Total	457	100	812	100	
Type of house					< 0.001
Masonry	10	2.3	46	5.6	
Processed wood	221	50.1	537	65.7	
*Paxiúba*	210	47.6	235	28.7	
Total	441	100	818	100	
Type of roof					< 0.001
Asbestos	55	12.2	26	3.2	
Zinc or aluminum	126	28.1	334	41.1	
Straw	268	59.7	452	55.7	
Total	449	100	812	100	
Type of floor					< 0.001
Wood	243	52.5	549	67.1	
Ceramics or cement	2	0.4	13	1.6	
*Paxiúb*a	218	47.1	256	31.3	
Total	463	100	818	100	
Number of rooms					< 0.001
1	50	10.8	171	21.2	
2	105	22.7	158	19.5	
3	307	66.5	480	59.3	
Total	462	100	809	100	
Introduction of cow’s milk					< 0.001
1-29 days	188	43.0	202	24.8	
30-730 days	74	16.9	570	69.9	
Never drank cow’s milk	175	40.1	43	5.3	
Total	437	100	815	100	
Place of birth					0.001
House	298	35.5	442	55.1	
Hospital	164	64.5	360	44.9	
Total	462	100	802	100	

Table 2 describes the transformations in health conditions that took place in Jordão between the surveys analyzed. A significant portion of households with children under five years had no toilet at home: 36.5% in 2005 and 34.8% in 2012. However, there was statistically significant improvement in the replacement of latrines for ceramic toilets in the houses between 2005 and 2012. In 65.6% of houses equipped with bathroom, this was built outside the house. This variable was only evaluable in 2012, since in 2005 it was not part of the questionnaire applied. Of the families that had no bathroom, 94.4% reported defecating in the backyard, bush, or open area and 5.6%, in the neighbors’ toilet.

**Table 2:** Sanitation conditions of houses with children under five years old living in the urban and rural area of the city of Jordão, Acre, 2005 and 2012.

Variable	Year of survey 2005		Year of survey 2012		p
	
	n	%	n	%	
Toilet					< 0.001
Absence	169	36.5	284	34.8	
Ceramic	99	21.4	276	33.8	
Wood	195	42.1	257	31.4	
Total	463	100	817	100	
Origin of water					
Rain	4	0.9	18	2.2	0.001
Public network	160	34.6	239	29.8	
Well or spring	76	16.4	90	11.2	
River	223	48.2	456	56.8	
Total	463	100	803	100	
Frequency of water shortage					
Never	335	72.5	504	62.1	< 0.001
Rarely	73	15.8	166	20.4	
Often	54	11.7	142	17.5	
Total	462	100	812	100	
Treatment of drinking water					< 0.001*
Boiled/Filtered and boiled	61	13.3	48	6.1	
Filtered	20	4.4	32	4.1	
Chlorinated at home	244	53.3	449	57.5	
Mineral	2	0.4	9	1.2	
Untreated	131	28.6	243	31.1	
Total	458	100	781	100	
Destination of trash					0.675
Collection	168	36.4	304	38.8	
Buried/Burned/Thrown into river	163	35.4	273	34.8	
Thrown in open area	130	28.2	207	26.4	
Total	461	100	784	100	
Destination of sewage					< 0.001
Septic tank	65	14.2	124	15.6	
Cesspit	130	28.4	317	39.8	
Open pit	210	46.0	276	34.7	
Others	52	11.4	79	9.9	
Total	457	100	796	100	

* Test did not include Mineral category.

Regarding the origin of water (Table 2) used in houses in 2005 and 2012, the use of river water predominated, representing 48.2% and 56.8%, respectively. More than a third of the houses did not present home connections of the public water supply in 2012. In 2005 and 2012, respectively, 72.5% and 62.1% of participants reported that water never lacks and 15.8% and 20.4%, that it rarely lacks. The chlorination of water at the houses was the treatment technique most reported by families with children under five in both surveys. A portion of the population uses water without any treatment before consumption, accounting for 31.1% of children under five years old. Concerning the destination of trash, there was no significant change, with predominance of public collection in 36.8% and 38.8% in 2005 and 2012, respectively. Regarding the destination of sewage, in 2005, 46.0% of the houses did it by open pit, 28.4% by cesspit, and 14.2% by septic tank. In 2012, cesspit and open pit were used by, respectively, 39.8% and 34.7% of houses.

Table 3 provides information about the presence of insects and rodents in the houses, as well as the care of families with food handling. Concerning flies, 28.0% of families classified as high the presence of this insect at home. The presence of cockroaches took place on 89.7% of houses, with 42.8% of families classifying as high the infestation of this insect. In relation to rodents, 78.8% reported presence, with 27.5% of houses showing high infestation. Regarding hygienic care during food handling, 88.7% reported washing their hands before handling food that will be consumed by children. Concerning the hygiene of fruits and vegetables eaten raw, only 23.8% of families washed them with treated water, 43.4% washed with untreated water, and 25.3% did not wash the fruits and vegetables consumed by children under five.

**Table 3:** Animal infestation and hygiene care in houses with children under five years old living in the urban and rural area of the city of Jordão, Acre, 2012.

Variable	Year of survey 2012	

	n	%
Insects and rodents		
Flies		
Not present	103	12.9
Low	357	44.8
Average	114	14.3
High	223	28.0
Total	797	100
Cockroaches		
Not present	84	10.3
Low	268	32.8
Average	115	14.1
High	349	42.8
Total	816	100
Rat		
Not present	162	21.2
Low	269	35.2
Average	123	16.1
High	210	27.5
Total	764	100
Washes hands when handling food		
Yes	708	88.7
No	90	11.3
Total	798	100
Care with raw fruits and vegetables		
Washed (treated water)	163	23.8
Washed (untreated water)	297	43.4
Does not wash	173	25.3
Does not consume raw fruits and vegetables	51	7.5
Total	684	100

Table 4 describes the association between diarrhea and the four determining factors selected: origin of water, type of house, age of introduction of cow’s milk, and place of birth for the years 2005 and 2012. About the origin of the water used, we identified, in both surveys, more probability of diarrhea among children who did not use water from the public supply network. In 2005, this risk was 1.38 times and in 2012, 1.60 times higher, when compared to children who used well water.

**Table 4:** Prevalence (%) and relative risk of diarrhea in children under five years old according to variables of origin of water, type of house, age of introduction of cow’s milk, and place of birth. Jordão, Acre, 2005 and 2012.

Variable	Year of survey 2005			Year of survey 2012		
	
	n	%	RR^b^	n	%	RR^c^
Water			p = 0.025			p = 0.024
Public network^a^	41	36.9	1	67	28.5	1
Well or river	114	51.1	1.38	14	60.9	1.6
Others	55	42.6	1.17	200	37.0	1.01
Type of house			p = 0.511			p = 0.081
Masonry or processed wood	107	43.7	1	187	32.1	1
*Paxiúba*	103	47.2	1.08	103	43.8	1.21
Age of introduction of cow’s milk			p = 0.022			p = 0.034
Never drank	71	40.6	1	10	23.3%	1
1-29 days	95	50.5	1.31	73	36.1	1.90
30-730 days	32	43.2	1.17	208	36.5	1.76
Place of birth			p = 0.031			p = 0.009
Hospital or maternity	83	50.6	1	101	28.1	1
House	126	42.3	0.78	185	41.9	1.40

^a^ Only in the urban area.

^b^ RR: Relative risk of diarrhea adjusted to the other variables of the Poisson multiple regression table.

^c^ Year 2005: water from well and without treatment.

In relation to the type of house, even not reaching statistical significance, this variable was kept in the model because of the significant changes observed in the construction of houses in the comparison between 2005 and 2012. This variable suggests higher probability of developing diarrhea in children living in households of *paxiúba*, which reached 1.21 times when compared to the value observed among children living in masonry or processed wood houses, in 2012.

In 2005, children receiving cow’s milk in the first month after birth showed probability of developing diarrhea 1.3 times higher than that of children who had never consumed cow’s milk and a probability 17.0% higher than the observed between those who received cow’s milk after 30 days. This same trend was observed in 2012, reaching twice the probability of occurrence of diarrhea episode of the first group and 1.76 times in relation to the second. Regarding the place of birth, in 2005, children born at home presented lower risk of diarrhea when compared to those who were born in hospital, with this difference reversing in 2012.

## DISCUSSION

In the seven years between the two surveys, we identified a high prevalence of diarrhea among children living in the city, but with a 9.7% reduction in the occurrence of this harm. Cesário et al.^3^, in a study of diarrhea prevalence in a teaching-health-care district of Rio Branco, Acre, in 2003, found prevalence of diarrhea of 33.3% in children under five years old. Benício et al.^2^ estimated the prevalence of diarrhea in children under five years of 10.5% in Brazil, of 12.5% in the North and, in São Paulo, of 4.7% considering the several socioeconomic strata and of 9.3% in children of underprivileged families. Vasquez et al.^14^ found 10.2% of prevalence of diarrhea in children of Pernambuco and 16.9% in the metropolitan region of Recife. Kariuki et al.^6^ (2012) reported that, in Turkana District, Kenya, the prevalence of diarrhea in 2007 was 43.7% and, in 2008, 30.7%. We observed that the prevalence of diarrhea in children of Jordão was superior to that of all researches mentioned.

Despite this high prevalence, we observed a reduction of this harm, at the same time with an increase in hospitalizations by diarrhea, between 2005 and 2012. Both situations can be a consequence of the implementation of the Family Health Program (PSF), in 2007, and of the inauguration of a Family hospital in the city, in 2008, which facilitated the access of the population to medium complexity care.

Jordão still has a rural population higher than the urban, which justify many houses not having electrical energy and, consequently, the advantages brought by this resource. This reality of the interior of the Amazon differs sharply from that experienced in the Center-South of Brazil, in which even small towns far from urban centers present an urban population greater than the rural and largest coverage of electrical energy services^1^.

Regarding social aid, the increase of families benefited by the Family Allowance Program was substantial, especially in the rural area of the city, increment factor for the acquisition of consumer goods or improving food quality.

We verified a change in the structure of houses, with increased use of processed wood for construction and floor, as well as on the number of rooms of the houses. This scenario shows improvement in the purchasing power of the population in this city and may indicate better infrastructure conditions of families with children under five, which certainly contribute to reducing exposures to diseases, including diarrhea.

As a way to evaluate the access to health services, we chose the variable of place of birth. The increase of home childbirth can be assigned to the fact that the population of the city is predominantly rural and, after the implantation of PSF, to increased prenatal care, training of midwives, and encouraging natural childbirth.

The introduction of cow’s milk on infant feeding is an important variable to evaluate the care and knowledge of the mother about the importance of breastfeeding. We observed late introduction of cow’s milk on infant feeding, decreasing the risk of diarrhea. These data indicate the importance of complementary feeding and breastfeeding practices for prevention of health problems in the early years of life. Cow’s milk is responsible for 20.0% of food allergies^7^; in addition, the digestion of the protein of cow’s milk is difficult in children, increasing the risk of diarrhea. The increase of the mother’s education level and PSF actions may have contributed to late introduction of cow’s milk on infant feeding.

A significant portion of the children still had no toilet in their homes, even with the construction of home health facilities in partnership with the *Fundação Nacional de Saúde* (FUNASA – National Health Foundation), which promoted the replacement of latrines for ceramic toilets in the houses between 2005 and 2012. There is a predominance of toilets outside the houses, which is typical of the rural area. This variable was only evaluable in 2012, since in 2005 it was not part of the questionnaire applied. In African countries, the use of latrine or outhouse is universalized^13^: approximately 92.4% in Uganda, 95.0% in Kenya, and 99.5% in Tanzania. The latrines in the urban area are built of permanent materials and, in rural areas, of mud and branches, to prevent fecal material from reaching the domestic environment^13^.

We observe the importance of the program for distribution of sodium hypochlorite in the city, since more than 50.0% of respondents rely on this resource for the treatment of drinking water. The Ministry of Health and State and Municipal Secretariats recommend the use of sodium hypochlorite solutions for disinfection of water in regions where there is no sanitation, because it is a simple, low cost, and effective preventive measure for fighting waterborne diseases^h^. The type of water treatment used in Jordão differs from other locations, such as in the city of Iporanga, São Paulo, where 69% of houses evaluated have piped water and, of these houses, 22.0% filter drinking water, 15.0% boil it, and 62.0% do not perform any treatment^5^.

Regarding the destination of trash, there was no improvement in collection and proper disposal in landfill between 2005 and 2012, a condition that contributes to the presence and proliferation of insects and rodents, which is sustained by the high presence of insects in the houses. In fact, the presence of flies is associated with infant diarrhea^11^.

Concerning the destination of sewage, in 2005, open pit and cesspit predominated, situations that favor the contamination of the environment near the houses and interfere in health conditions of children^10^. The lack of sewage disposal system interferes directly in children health for polluting the environment and allowing the transmission of excreta-related diseases, mainly parasitic ones. The disposal of excreta in open land or street is a risk for helminthiasis and, consequently, for other waterborne diseases, as diarrhea^i^.

We verified the care with the hygiene of hands before food preparation, but a small portion did not perform such care, contributing to the risk of contamination during food preparation. Regarding fruits and vegetables consumed raw, an expressive portion washed them, but with untreated water. As the water came predominantly from rivers, this practice can offer risk of disease transmission, especially diarrhea. The water used for consumption in rural and urban areas, in this study, came from well or river, representing a 38.0 and 60.0% higher risk (in 2005 and 2012) for diarrhea than the consumption of water from the public network. However, the risks found for diarrhea were lower than those found by Teixeira and Heller^11^ in areas of subnormal settlement in the city of Juiz de Fora. Washing hands with water and soap can reduce 48.0% of the risk of diarrhea; improving water quality, 17.0% of risk; and proper sewage collection, 36.0% of risk^4^.

The sanitation conditions in the city presented an expressive improvement, with increase in the number of bathrooms with toilets, implementation of water treatment in the urban area of the city, increased maternal education, and access to health services. The multivariate regression model identified a statistically significant association between use of water from the public network, type of house, late introduction of cow’s milk, and access to health service with occurrence of diarrhea. Although the sanitary transformations that took place in Jordão were important on the occurrence of diarrhea, water supply and sewage systems remain not universalized. Expansion must be prioritized, since this service has not yet achieved a large portion of the population living in rural areas.

## Footnotes

a Brasil. Law no. 11,445, of January 5, 2007. Establishes national guidelines for basic sanitation; changes Laws no. 6,766 (December 19, 1979), 8,036 (May 11, 1990), 8,666 (June 21, 1993), 8,987 (February 13, 1995); repeals Law no. 6,528 (May 11, 1978); and creates other measures. *Diario Oficial Uniao*. 11 jan 2007.

b Teixeira JC. Associação entre cenários de saneamento e indicadores de saúde em crianças. Estudo de áreas de assentamento subnormal em Juiz de Fora, MG [these]. Belo Horizonte (MG): Escola de Engenharia da UFMG; 2003.

c World Health Organization (WHO). Diarrhea: why children are still dying and what can be done. Geneva: World Health Organization; 2009 [cited 2014 Oct 31]. Available from: http://apps.who.int/iris/bitstream/10665/44174/1/9789241598415_eng.pdf

d Brasil. Constituição da República Federativa do Brasil (1988). Brasília (DF): Senado; 1988.

e Instituto Brasileiro de Geografia e Estatística. Censo demográfico 2010 [cited 2013 Aug 14]. Available from: http://www.ibge.gov.br/home/estatistica/populacao/censo2010

f Atlas do Desenvolvimento Humano no Brasil. Índice de desenvolvimento humano municipal (IDHM). Brasília (DF): PNUD; 2013.

g Popular name of a palm of the *Arecaceae* family; it occurs in Central and South America and in Brazil, and it is used for building houses and making floors.

h Ministério da Saúde (BR). Indicadores de morbidade e fatores de risco. Brasília (DF): Ministério da Saúde; 2006.

i Teixeira JC; Heller L. Fatores ambientais associados às helmintoses intestinais em áreas de assentamento subnormal, Juiz de Fora, MG. *Eng. Sanit.Ambient.* 2004;9(4),301-5. DOI:10.1590/S1413-41522004000400006
